# The Introduction of a Protocol for the Use of Biobrane for Facial Burns in Children

**DOI:** 10.1155/2011/858093

**Published:** 2011-09-27

**Authors:** A. D. Rogers, S. Adams, H. Rode

**Affiliations:** ^1^Burns Unit, Red Cross War Memorial Children's Hospital, Klipfontein Road, Rondebosch, Cape Town 7700, South Africa; ^2^Division of Plastic Surgery, Groote Schuur Hospital and Red Cross War Memorial Children's Hospital and The University of Cape Town, Cape Town, South Africa; ^3^Division of Paediatric Surgery, Red Cross War Memorial Children's Hospital and The University of Cape Town, Cape Town, South Africa

## Abstract

Biobrane has become an indispensible dressing with three established indications in acute burns care at our institution: (1) as the definitive dressing of superficial partial thickness facial burns, (2) after tangential excision of deep burns when autograft or cadaver skin is unavailable, and (3) for graft reduction. This paper details our initial experience of Biobrane for the management of superficial partial thickness facial burns in children and the protocol that was compiled for its optimal use. A retrospective analysis of theatre records, case notes and photographs was performed to evaluate our experience with Biobrane over a one-year period. Endpoints included length of stay, analgesic requirements, time to application of Biobrane, healing times, and aesthetic results. Historical controls were used to compare the results with our previous standard of care. 87 patients with superficial partial thickness burns of the face had Biobrane applied during this period. By adhering to the protocol we were able to demonstrate significant reductions in hospital stay, healing time, analgesic requirements, nursing care, with excellent cosmetic results. The protocol is widely accepted by all involved in the optimal management of these patients, including parents, anaesthetists, and nursing staff.

## 1. Background

Biobrane was conceived in 1979 but has only been available in South Africa since 2007. It is a bilaminar material composed of an outer silicone film with partially embedded nylon. Porcine type one collagen is incorporated into both components. The collagen peptides bind to the wound surface fibrin and so act as a dermal analogue. Re-epithelialisation is thus facilitated which results in spontaneous detachment of the Biobrane once complete. Due to the presence of pores in the product, exudate may drain, and antimicrobial dressings (like Acticoat) may be applied to the wound surface. 

Systematic reviews have been completed relating to the use of bioengineered skin substitutes. The use of Biobrane has not only yielded excellent results in the management of partial thickness burns but has also been used with success as a temporizing dressing after excision of full-thickness and deep dermal burns (particularly when cadaver allograft is unavailable) and for so-called “graft reduction” for burns of equivocal depth. In addition, promising results have been seen in the management of toxic epidermal necrolysis, paraneoplastic pemphigus, chronic wounds, donor sites, and after dermabrasion and laser resurfacing [1–4].

For partial thickness burns in adults, several studies have demonstrated reductions in hospital stay, healing times, dressings, pain scores, and overall treatment costs, without increased infection rates, when compared to silver sulphadiazine or nonadherent dressings like tulle gras [1, 2].

There is, however, a paucity of literature on the use of Biobrane for superficial partial thickness facial burns in children, particularly in those with black skin, in whom changes in pigmentation on the face can have devastating cosmetic sequelae.

Facial scalds comprise as many as 30% of admissions to the paediatric burns unit at the Red Cross War Memorial Children's Hospital in Cape Town. The vast majority of these injuries are partial thickness depth and heal within three weeks with traditional dressing care. The minority develop complications; these can have devastating complications.

According to Herndon et al., “Partial thickness burns in children have been treated for years by daily, painful tubbing, washing and cleansing, followed by topical application of antimicrobial creams awaiting spontaneous healing” [2].

This study aimed to evaluate our experience with Biobrane for superficial partial thickness facial burns in children and to design and implement a protocol for its optimal use.

It should be emphasized that a large number of so-called skin substitutes other than Biobrane are in use for the management of burn wounds. However, neither Integra nor Matriderm is available in South Africa at this time, despite their remarkable potential to “remodel” the dermis prior to reepithelialisation. This study identifies the uses of Biobrane and emphasizes the protocol introduced for its main indication in our setting, namely as a temporary skin substitute for superficial partial thickness burns (similar to its value as a donor-site dressing). 

Integra (Integra LifeSciences Corp., Plainsboro, NJ, USA) is a dermal regeneration template consisting of bovine collagen, chondroitin-6-sulphate, and a silastic membrane. Of note, its value has been in the treatment of deep partial thickness and full-thickness burn wounds, full-thickness skin defects of different aetiologies, chronic wounds, and in soft tissue defects, rather than in the wounds described in this study. 

Matriderm (Skin and Health Care AG, Billerbeck, Germany) is a structurally intact matrix of bovine type I collagen with elastin with similar indications to Integra, although it provides added benefits in terms of elasticity and skin graft take.

## 2. Materials and Methods

A retrospective case note review was performed on patients with superficial partial thickness facial burns of the face who were admitted to the Burns Unit and had Biobrane applied between August 2008 and July 2009.

The Burns unit at the Red Cross War Memorial Children's Hospital admits children under the age of 13 years.

Demographic details, mechanism of injury, features of treatment implemented, and endpoints were examined. Endpoints were length of stay, time to theatre, analgesic requirements, time to healing, and initial aesthetic results.

Outcomes were compared with 20 matched historical controls.

Exclusion Criteria are

1. haemodynamically unstable patients,
2. those who could not have Biobrane applied within 48 hours,
3. Those admitted after 24 hours of the burn injury,
4. other special areas involved (e.g., hands), which may result in prolonged hospitalization,
5. more than 20% body surface area burns.

Standard burns protocols were applied, including intravenous and enteral resuscitation, and early excision and grafting, when indicated. 

Departmental and institutional approval was obtained for this study.

## 3. Results

87 patients with superficial partial thickness burns of the face were studied. 50 (57%) of these patients were male, and the mean age of the patients was 3.2 years (range 0.5 to 12).

81 (93%) of the patients sustained scalds and the remainder flash flame burns. The mean total body surface area of the burn wound was 6% (range 1–20%).

The mean hospital stay of the control group (matched for age and total body surface area burnt) was 6 days. During the first 6 months of the period in question (i.e., August 2008 to January 2009), the mean hospital stay for the Biobrane group was 4.8 days, whereas during the last 6-month period (i.e., February to July 2009) the mean hospital stay was reduced to 3.5 days. 

The mean healing time of the superficial partial thickness facial burns was 12 days in the control and 10 days in the Biobrane group.

Opiate Analgesia used for the Biobrane group was one-third of that used in the control group. This was largely due to reduced procedural rather than baseline analgesic requirements.

There was one case of infection in the control group (5%), and 5 in the Biobrane group (5.7%). *Pseudomonas aeruginosa* was cultured in 3 of these cases, and all appeared to originate from the ear. It has become policy for the unit to apply antimicrobial ear drops (chloromycetin) perioperatively. There were two cases where *Staph aureus* was cultured. 2 of these cases required debridement and skin grafting.

Although not a primary endpoint in the study, there appeared to be a trend towards reduced mixed and hypopigmentation in those managed with Biobrane, as compared to the standard therapy group.

A protocol was drawn up and implemented consistently thereafter (Table 1).

## 4. Discussion

Biobrane is now an indispensible dressing in the burns surgeon's armamentarium. Although no perfect dressing exists for all burn injuries, Biobrane has been shown to be the superior option in three distinct settings within the context of acute burns care as follows:

1. for dressing superficial partial thickness burns, particularly of the face,
2. after tangential excision of deep dermal and full-thickness burns, when cadaver skin or autograft is unavailable or insufficient,
3. for graft reduction, that is, in areas where depth is equivocal, the use of Biobrane may reduce the necessity for skin grafting.

This study identifies the caveats for the successful use of Biobrane for the first indication, where it is the definitive dressing. In these cases, Biobrane facilitates spontaneous healing of the superficial partial thickness facial burn. Its use has been shown to reduce the use of procedural opiate analgesia and hospital length of stay.

We hypothesise that more rapid healing of the facial burn is facilitated by a single definitive dressing rather than by daily or alternate day dressings that would be required by standard dressing choices, for example, silver sulphadiazine or tulle gauze. There is likely to be less interference in the reepithelialisation of the superficial partial thickness burn wound.

**Figure 1:**
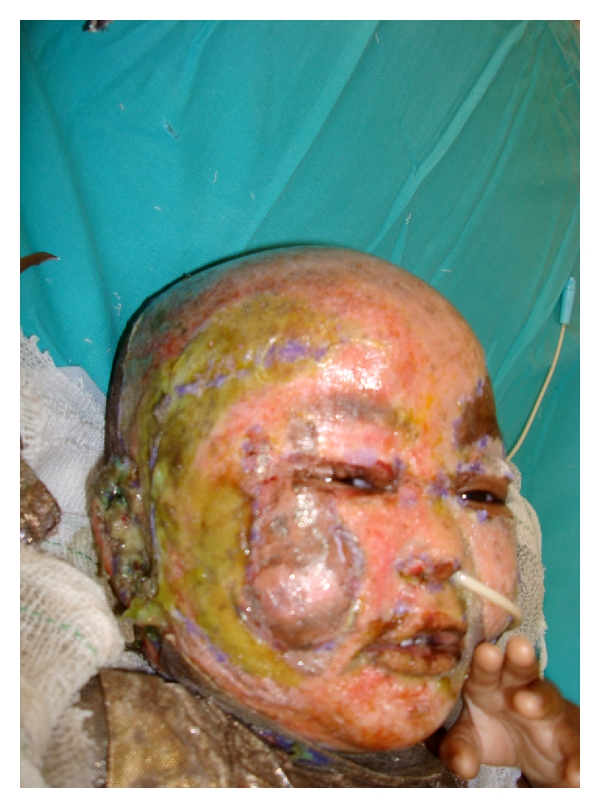
*Pseudomonas* Infection of the Biobrane, in a deep partial thickness facial burn.

Adherence to strict protocols is essential to reduce the incidence of complications, particularly nonadherence, and infection (Table 1). We recommend restricting its use to the first 48 hours after the burn injury, as the incidence of infection escalates dramatically thereafter. In our practice, several infections appear to have arisen from presumed carriage in the ears, and chloromycetin is now routinely applied perioperatively (Figure 1). 

In the paediatric setting, Biobrane application is usually performed in theatre, with the child under general anaesthetic. 

Application for this indication should be restricted to suitable facial burns, preferably unequivocally superficial partial thickness depth. The clinician should preferably observe blanching and blistering, which both imply the presence of an intact subdermal circulation. In these cases, failure of adherence is rare. Nonadherence and subsequent infection is far more prevalent in deeper nonexuding burn injuries (Figure 1). 

**Figure 2:**
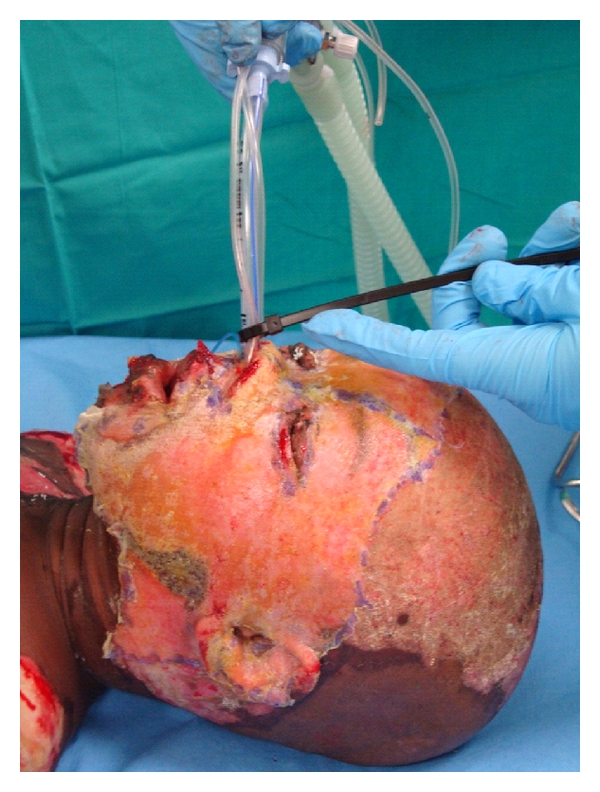
Following application of Biobrane, using the blue Butyl-2-cyanolacrylate, around the edges, and the Gray-Rode method to secure the endotracheal tube.

We have found the so-called Gray-Rode technique particularly effective to secure the endotracheal tube and remove any ties from the operative field (Figure 2). A nasogastric tube is looped around the hard palate and secured to the endotracheal tube with a cable tie. An adaptation of this method may also be utilized for patients intubated via the nasal route [5].

The face should then be thoroughly cleaned with an antimicrobial agent, followed by saline. Blisters should be removed. Versajet on a low setting has been effectively used, although is not always necessary.

Biobrane should then be applied, according to the manufacturers' instructions, dull side down, at a stretch. A single piece is preferable to reduce seams and consequent scarring. If necessary, seams should be placed between aesthetic subunits. 

We have found the application easily performed by a single surgeon with the use of Histoacryl or Dermabond. These products are butyl-2-cyanoacrylate, a rapid acting adhesive. The application of just a few drops allows one to stretch the Biobrane over the contours of the face almost instantaneously (Figure 2). There is evidence that these agents may also contribute a bacteriostatic effect at the edge of the Biobrane [6]. This product also significantly reduces the operative time when compared to alternatives like adhesive tapes, strips, and sutures. Adhesive materials are also difficult to apply effectively over moist wounds and near or in the hair. Staples should be avoided on the face for two reasons; they are painful to remove and may leave obvious scars if left in situ for more than seven days.

Antimicrobial dressings may be applied according to surgeon's preference, and the wound covered. We have found several dressings, including nanocrystalline silver (Acticoat) and povidone iodine-based dressings (Inadine), to assert their activity in the pores of the Biobrane bilayer. 

Postoperatively, all patients, particularly those younger than 5 years, are commenced with nasogastric feeds. We have found this to improve the adherence of the Biobrane around the mouth and nose.

**Figure 3:**
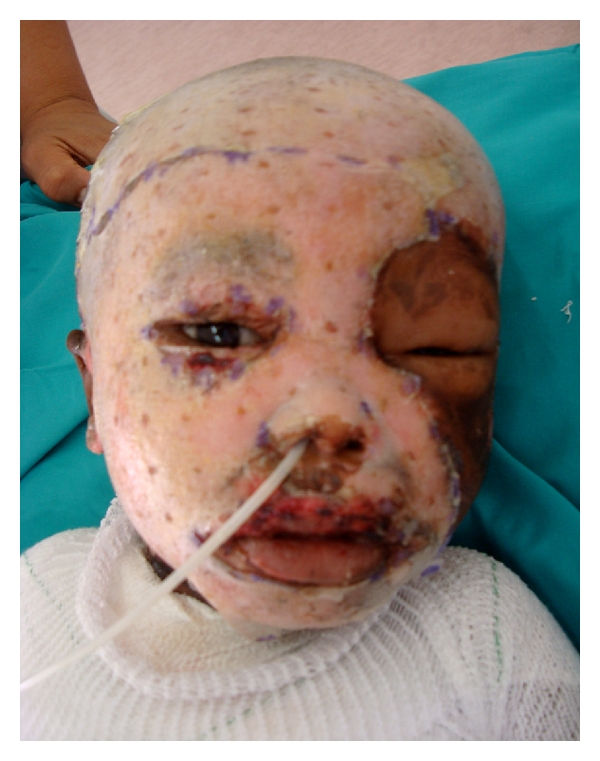
At Inspection 48 hours after application of Biobrane, which is completely adherent to the burn wound.

**Figure 4:**
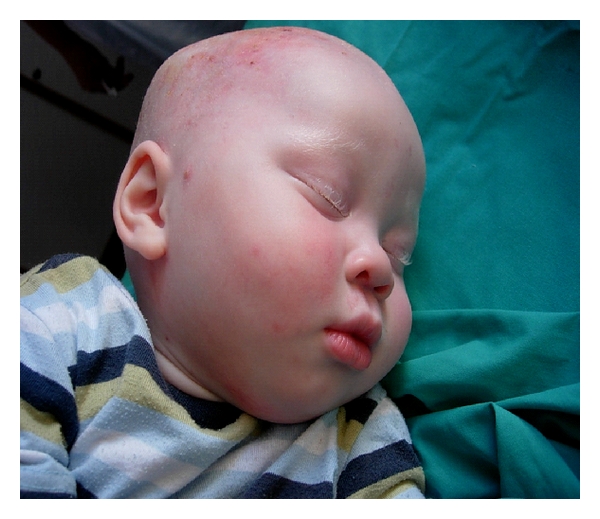
After 14 days of Biobrane application in an Albino baby.

Overlying dressings are removed after 48 hours and inspected (Figure 3). If adherent, many of our patients may be considered for discharge. If not adherent, the Biobrane would be trimmed or removed if there are features of infection. Thereafter the Biobrane is covered and reviewed periodically until reepithelialisation occurs, which is followed by separation (Figure 4). During the second 6-month period of the study, we were able to further reduce the hospital stays, as early discharge appeared possible, even to very underprivileged homes.

Although per unit, in Biobrane is an expensive dressing option, its costeffectiveness is demonstrated when one considers the reduced healing times, hospital stay, and nursing staff time allocated to dressing changes. Procedure-based analgesia has also been significantly reduced. 

Biobrane is now readily applied after hours and on weekends if necessary, and there is a recognition by all involved in the care of these children of its efficacy, including parents, ward and theatre staff, and anaesthetists. A few Muslim parents have consented to the use of Biobrane, partially composed of porcine collagen, for their children. 

**Figure 5:**
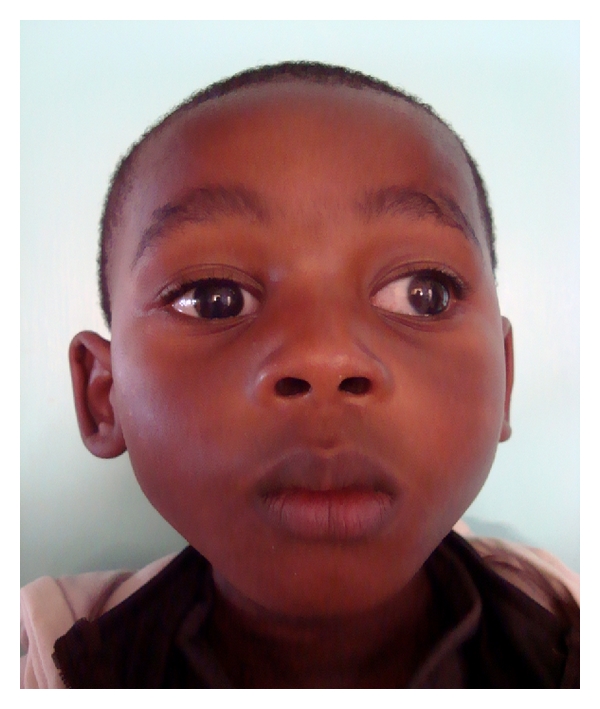
One month after Biobrane.

This protocol is now integrated into the care of patients with superficial partial thickness burns of the face (Table 1). More than one hundred similar cases are performed annually at our unit with few complications. These children can expect shorter hospital stays, reduced healing times, dressing changes, and pain experienced, and probably improved aesthetic results, particularly with respect to altered pigmentation. Black children comprise the majority of our patients (Figure 5). Further prospective studies are underway to determine the final aesthetic results of these children.

## Figures and Tables

**Table 1:** The Red Cross War Memorial Children's Hospital Burns Unit.

Protocol: Biobrane for Superficial Partial Thickness Facial Burns
(1) Select a suitable burn
(a) Superficial partial thickness
(i) Blistering
(ii) Blanching
(b) Senior review
(2) Apply within 48 hours of burn
(a) Limit infection
(b) Theatre and staff availability
(c) General anaesthetic
(3) No contraindications
(a) Burn related
(b) Comorbid conditions
(c) Haemodynamically stable
(d) Use of porcine product
(4) Use Gray-Rode Technique
(a) Endotracheal tube Stabilisation
(i) Nasogastric tube and cable tie
(ii) Looped around hard palate
(b) Reference [5]
(5) Clean well
(a) Chlorhexidine or Betadine
(b) Saline
(c) Versajet if required
(d) Chloromycetin ear drops
(6) Apply at stretch
(a) Dull side down
(b) Single piece preferably
(c) Reduce seams
(d) Histoacryl/Dermabond/plasma to secure
(7) Dressing and cover
(8) Nasogastric tube feeds
(9) Review at 48 hours
(a) Trim if not adherent
(b) Remove if infected
(10) Await spontaneous separation/healing

## References

[1] Lal S, Barrow RE, Wolf SE (2000). Biobrane^®^ improves wound healing in burned children without increased risk of infection. *Shock*.

[2] Barret JP, Dziewulski P, Ramzy PI, Wolf SE, Desai MH, Herndon DN (2000). Biobrane^®^ versus 1% silver sulfadiazine in second-degree pediatric burns. *Plastic and Reconstructive Surgery*.

[3] Whitaker IS, Prowse S, Potokar TS (2008). Critical evaluation of the use of Biobrane^®^ as a biologic skin substitute: a versatile tool for the plastic and reconstructive surgeon. *Annals of plastic surgery*.

[4] Pham C, Greenwood J, Cleland H, Woodruff P, Maddern G (2007). Bioengineered skin substitutes for the management of burns: a systematic review. *Burns*.

[5] Gray R, Rode H (2010). Intra-operative endotracheal tube stabilisation for facial burns. *Burns*.

[6] Bhende S, Rothenburger S, Spangler DJ, Dito M (2002). *In Vitro* assessment of microbial barrier properties of Dermabond^®^ topical skin adhesive. *Surgical Infections*.

